# Reviving low-affinity glycan-targeting CAR with combinatorial design

**DOI:** 10.1016/j.xcrm.2026.103108

**Published:** 2026-10-08

**Authors:** Christopher Forcados, Hakan Köksal, Eveline Scherer, Rafaela Abrantes, Pierre Dillard, Henrique O. Duarte, Sarah Josefsson, Catarina Gomes, Constance de Montlaur, Henrik T.Z. Hellesøy, Ryan Dunkel, Erlend.B. Smeland, June H. Myklebust, Celso A. Reis, Emmet McCormack, Pascal Gelebart, Else Marit Inderberg, Sébastien Wälchli

**Affiliations:** 1 Translational Research Unit, Section for Cellular Therapy, Department of Oncology, Oslo University Hospital, 0379 Oslo, Norway; 2 Faculty of Medicine, University of Oslo, 0316 Oslo, Norway; 3 i3S, Institute for Investigation and Innovation in Health, University of Porto, 4200-135 Porto, Portugal; 4 IPATIMUP – Institute of Molecular Pathology and Immunology, University of Porto, 4200-135 Porto, Portugal; 5 Department of Cancer Immunology, Institute for Cancer Research, Oslo University Hospital, 0310 Oslo, Norway; 6 Department of Clinical Science, Precision Oncology Research Group, University of Bergen, 5020 Bergen, Vestland, Norway; 7 Centre for Cancer Biomarkers (CCBIO), University of Bergen, 5020 Bergen, Vestland, Norway; 8 Centre for Pharmacy, Department of Clinical Science, University of Bergen, 5020 Bergen, Norway; 9 Department of Haematology, Haukeland University Hospital, 5020 Bergen, Vestland, Norway; 10 Faculty of Mathematics and Natural Sciences, University of Oslo, 0315 Oslo, Norway

**Keywords:** Chimeric antigen receptor T cells, glycan, CD75s, Combinatorial CAR, CAR T cell therapy, immunotherapy, α2,6-linked sialic acid, B cell lymphoma

## Abstract

CD19CAR T cells demonstrate remarkable therapeutic efficacy in the treatment of B cell malignancies. A global limitation of conventional CD19CAR therapy is its mono-specificity. Thus, defining complementary targets to CD19 is critical to avoid target-dependent limitations. Alternatives to protein antigens include secondary modifications, such as glycosylation. One example of aberrant glycosylation in lymphoma is the increase in *N*-glycans capped with α2,6-linked sialic acid, named CD75s. Here, we generate a CAR that recognizes CD75s based on an IgM monoclonal antibody called HH2. HH2CAR shows cytotoxic activity against B cell lymphoma cell lines, although it induces little to no cytokine release. However, while HH2CAR alone cannot control tumor progression *in vivo*, its combination with CD19CAR results in a functional molecule. Beyond overcoming the limited efficiency of HH2CAR T cells, this combination also prevents potential off-tumor/on-target toxicity associated with CD19 single targeting. Thus, inefficient mono-specific CAR molecules can be effectively leveraged in combinatorial formats.

## Highlights

• α2,6-linked sialic acid (CD75s) is a potential CAR target against B cell lymphoma
• HH2CAR T cells recognize CD75s-positive target cells but exhibit weak performance
• Functionality of low-affinity HH2CAR can be rescued in combinatorial CAR design

## Graphical abstract

**Figure:**
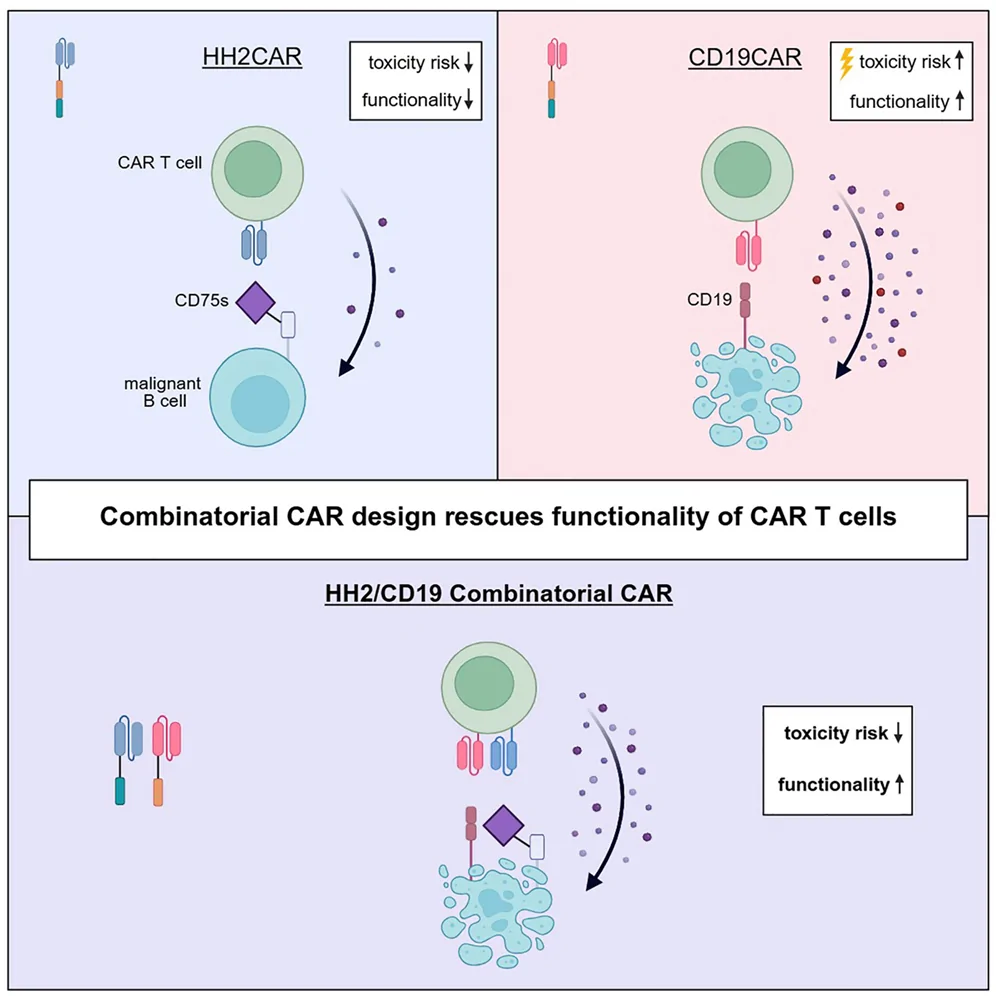

## Teaser

Forcados et al. develop and test a combinatorial CAR strategy against B cell lymphoma, in which they combine CD75s and CD19 targeting. This combinatorial approach overcomes the limited efficiency of CD75s mono-targeting and lowers the risk of CAR-related toxicity seen with CD19 single targeting.

## Introduction

Adoptive cell therapy (ACT) based on chimeric antigen receptor (CAR) T cells has been widely developed during the past years. Efficient and safe CAR T cell therapy relies on identifying suitable cancer-specific molecular targets, which ideally should be restricted to tumor cells. However, achieving true cancer restriction has proven to be an outstanding challenge. For example, CD19, which remains the most efficient and widely exploited target since it is expressed in over 95% of B cell malignancies (e.g., B cell acute lymphoblastic leukemia [B-ALL], chronic lymphoid lymphoma [CLL], and B cell non-Hodgkin lymphoma [B-NHL]), is also expressed on healthy B cells.^1^^,^^2^^,^^3^^,^^4^ Thus, CD19CAR T cell therapy carries the risk of developing B cell aplasia, which, although considered clinically manageable by many,^5^ is associated with shorter overall survival due to infection-related deaths.^6^ Infection as the main cause of non-relapse death, cytokine release syndrome (CRS),^7^ and the recent report of CD19 expression in brain mural cells,^8^ which may potentially be linked to the neurotoxicity observed in CD19CAR clinical trials,^9^ are the main concerns of CD19CAR T cell therapy. These toxicities challenge the concept of restricted targeting and suggest that alternative CAR targets might be needed.

With the intention of identifying an alternative CAR to CD19CAR, we produced a CAR molecule derived from a previously isolated monoclonal antibody (mAb), HH2, chosen for its preferential binding to human malignant B cells.^10^ Indeed, HH2 targets the α2,6-sialylated CD75 *N*-glycan antigen (CD75s),^11^ which is significantly enriched in several liquid and solid malignancies.^12^^,^^13^^,^^14^ Initially, HH2 was characterized as restricted to human B cells, targeting a sialylated epitope, CD75s, generated by the oncogenic sialyltransferase β-galactoside α2,6-sialyltransferase 1 (ST6Gal1).^11^^,^^15^^,^^16^^,^^17^ Beyond B cells, CD75s has also been investigated in gastric,^18^^,^^19^ breast,^20^ and colorectal^21^ carcinomas. We first characterized the ability of CD75s-targeting CAR T cells (HH2CAR T cells) to induce specific cytotoxic activity against B cell lymphoma cell lines. However, production and release of cytokines upon antigen encounter was minimal, and consequently, HH2CAR alone did not control tumor progression *in vivo*. Our attempts to modify the signaling tail design were unsuccessful in improving its potency.

The CAR molecules most commonly used in the clinic are the so-called second-generation CARs, where the intracellular signaling domain is composed of signal 1 (CD3ζ) and signal 2 (CD28 or 4-1BB) components. In contrast to first-generation CARs, carrying only signal 1, second-generation CARs provide T cells with the power to both kill and persist in the body. Modifications of the extracellular part have also been proposed. Dual CARs with two different single-chain fragment variables (scFvs) placed on the same molecule have been shown to improve cancer cell recognition and overcome antigen escape and have recently been tested in clinical trials.^22^ We^23^ and others^24^ previously proposed an alternative strategy in which the binding domains are physically separated, with one fused to a signal 1 (CD3ζ) cytoplasmic domain and the other to signal 2 (4-1BB). This combinatorial design is expected to restrict T cell activation in settings where only one of the two antigens is encountered. In CAR T cell therapy against B cell malignancies, CD19CAR T cells are highly potent and induce strong activation of the engineered T cells. We tested whether the combinatorial CAR design could potentiate HH2CAR activity by adding CD19 targeting. Importantly, our previously described combinatorial design^23^ accommodated the HH2 scFv. In more detail, this design creates two separate CAR molecules: the HH2 scFv linked to CD3ζ, delivering signal 1, and the CD19-targeting scFv (Fmc63 clone) linked to 4-1BB to achieve a supporting signal upon CD19 recognition.

In the present study, we evaluated the efficacy of this combinatorial-targeting approach to enhance CD75s targeting. HH2/CD19CAR demonstrated potent and more restricted activation. Additionally, the combinatorial design increased *in vitro* functional avidity and cytokine production and significantly controlled tumor growth *in vivo*. By overcoming the limitations of less potent CAR T cell therapies and increasing target restriction, this strategy provides a valuable resource for advancing CAR T cell design and effectiveness against B cell malignancies.

## Results

### ST6Gal1-dependent CD75s antigen expression in B-NHL

We assessed CD75s antigen expression by measuring binding of the HH2 mAb on patient samples from various B-NHL subtypes. High expression was detected on tumor cells of follicular lymphoma (FL), mantle cell lymphoma (MCL), and marginal zone lymphoma (MZL/CLL). Interestingly, tumor cells of diffuse large B cell lymphoma (DLBCL) samples showed a more heterogeneous expression pattern for CD75s (Figure 1A). Several lymphoma cell lines were analyzed by flow cytometry, including cells derived from Burkitt lymphoma (BL), DLBCL, FL, and MCL, together with two leukemia cell lines, K562 and J76, used as controls. Both HH2 and anti-CD19 antibodies were used for staining (Figures 1B and 1C). All malignant B cell lines, except MAVER-1 derived from MCL, showed a similar percentage of CD75s-positive cells (>90%, Figure 1B). Yet, they displayed varying fluorescence intensities for CD75s and CD19 staining relative to those for BL-41 (Figure 1C). As BL-41 had uniformly high expression levels of HH2 and CD19, we performed most of the following experiments using this cell line. To further assess the localization of CD75s, we performed fluorescence microscopy imaging of BL-41 and Caco-2 cells with the HH2 mAb (Figure 1D). CD75s was stained both intracellularly and at the membrane of these cells. Upon neuraminidase treatment to cleave sialic acid molecules, CD75s recognition by the HH2 mAb was completely lost at early time points, with expression slowly starting to recover after 6 h, and at 24 h, it did not reach total recovery to the basal level of CD75s detection by the HH2 mAb. This confirms that HH2 recognizes a sialylated epitope. In contrast, CD19 detection was not affected by neuraminidase treatment (Figure 1E). We further analyzed the expression of CD75s in peripheral blood mononuclear cells (PBMCs) given prior observations of a small subset of T cells and monocytes expressing CD75s.^25^ Among the four main immune cell subsets, B cells were the only cells that were strongly positive for CD75s (Figure 1F), although at a lower level than their malignant counterparts (Figure 2D). *ST6GAL1* knockout (KO) in BL-41 resulted in loss of HH2 staining (Figure S1A). Altogether, these results demonstrate that the HH2 mAb recognizes the terminal sialylated CD75s antigen, generated by ST6Gal1, and specifically stains a wide range of B-NHL patient samples and cell lines, thus being a relevant candidate for CAR T cell therapy.

**Figure 1:**
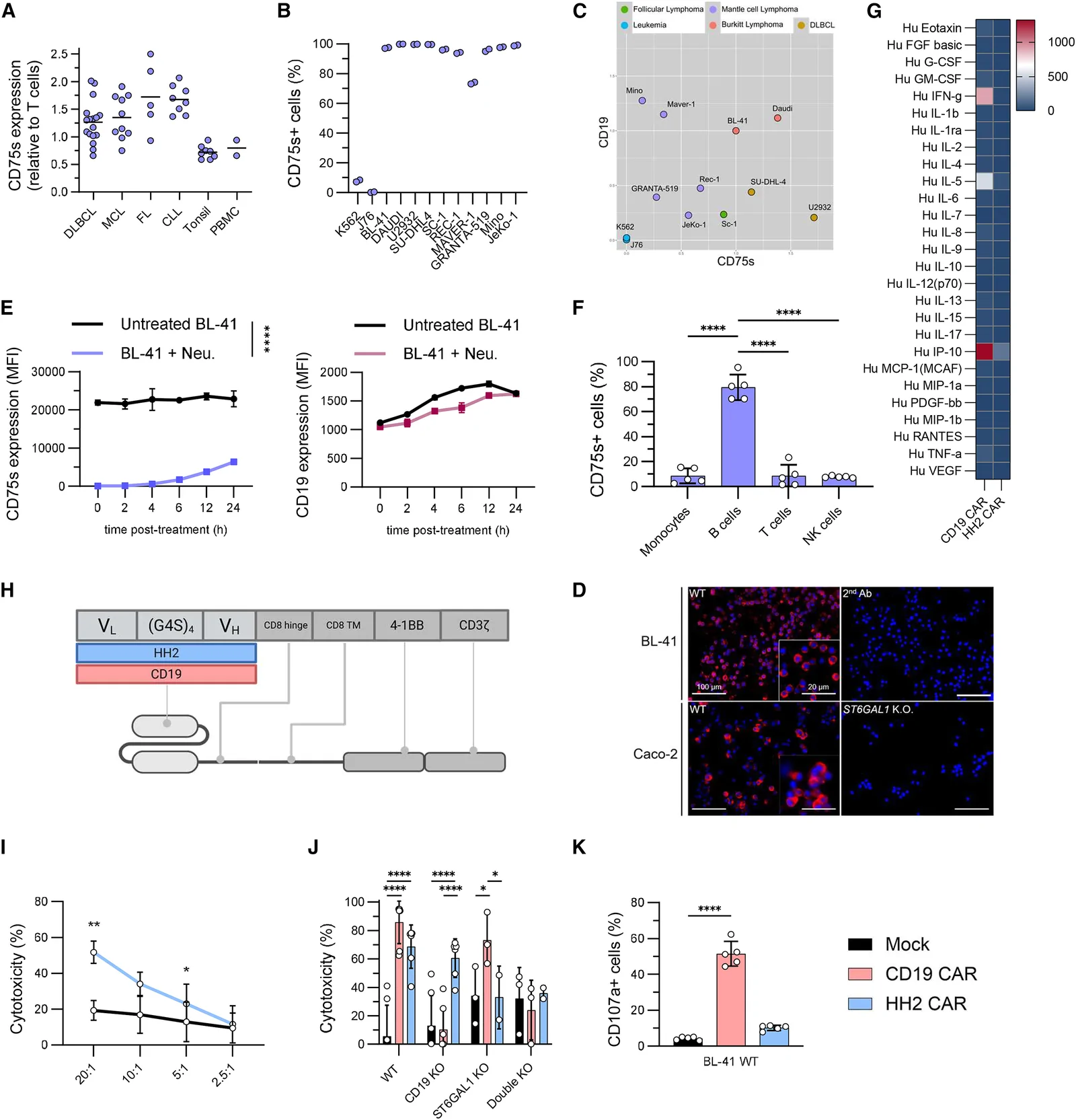
Characterization of the anti-CD75s antibody (clone HH2) in B cell malignancies and its potency as a CAR (A) Flow cytometry (FCM) detection of the surface expression of CD75s with HH2 clone in patient samples of follicular lymphoma (FL) (*N* = 5), diffuse large B cell lymphoma (DLBCL) (*N* = 17), mantle cell lymphoma (MCL) (*N* = 10), chronic lymphoid lymphoma (CLL) (*N* = 8), B cells from healthy donor PBMCs (*N* = 2), and tonsils (*N* = 8). Tumor cells or normal B cells were identified as CD3^−^CD20^+^, and the expression is relative to CD3^+^CD20^−^ T cells in the same sample. Each dot represents a separate patient. (B) Percentage of CD75s^+^ cells, obtained by FCM, across different cell lines. *n* = 2 technical replicates. Values plotted are the mean, and the error bars represent SD. (C) Scatterplot representing the fluorescence intensity of CD19 and CD75s surface staining of several cell lines relative to BL-41 (BL) cells assessed by FCM (*n* = 2 technical replicates). (D) Fluorescence microscopy of BL-41 and Caco-2 cells stained with HH2 (in red) and DAPI (in blue). Scale bars indicate 100 μm in the overview images and 20 μm in the magnified images. (E) FCM staining of BL-41 cells at indicated time points after treatment with neuraminidase (Neu.) and without Neu. treatment. Cells were stained with HH2 (left) or CD19 (right). Fluorescence intensity is plotted. *n* = 2; the error bars represent SD; two-way ANOVA was used for statistics. (F) Percentage of CD75s-positive cells among the different PBMC subsets. *N* = 5 healthy donors; the error bars represent SD; one-way ANOVA was used for statistics. (G) BioPlex assay (27-plex) of cytokine production from the supernatant of T cells either transduced with CD19CAR or HH2CAR co-cultured with BL-41 cells for 24 h. Fold change of cytokines produced compared to mock T cells is displayed. *N* = 1 healthy donor; *n* = 3; mean values are plotted. (H) Representation of the second-generation CAR design of CD19 or HH2CAR. DNA sequence depicting an scFv of CD19 (red) and HH2 (blue), consisting of a variable light (V_L_) and variable heavy (V_H_) chain, which is linked by a (G_4_S)_4_ linker, CD8-derived hinge and transmembrane (TM) domain, 4-1BB-derived costimulatory domain, and CD3ζ-derived signaling domain. (I) BLI-based cytotoxicity assay against BL-41 cells after 6 h with T cells transduced with HH2CAR (in blue) or mock T cells (black) at indicated E:T ratios. *N* = 3 healthy donors; the error bars represent SD; two-way ANOVA was used for statistical analysis. (J) BLI-based cytotoxicity assay against WT, CD19 KO, *ST6GAL1* KO, or double KO BL-41 cells with T cells either transduced with CD19CAR (in red) or HH2CAR (in blue), or non-transduced (mock, in black). Percentage of specific cell lysis at the 4 h time point is plotted. *N* = 2–7 healthy donors; the error bars represent SD; two-way ANOVA was used for statistics. (K) CD107a degranulation assay against BL-41 cells with T cells either transduced with CD19CAR (in red) or HH2CAR (in blue), or non-transduced (mock, in black). Percentage of CD107a-positive cells is plotted. *N* = 5 healthy donors; the error bars represent SD; one-way ANOVA was used for statistics. *p* values are depicted as ∗∗∗∗*p* < 0.0001, ∗∗∗*p* < 0.001, ∗∗*p* < 0.01, and ∗*p* < 0.05, and *p* > 0.05 are non-significant (not shown).

**Figure 2:**
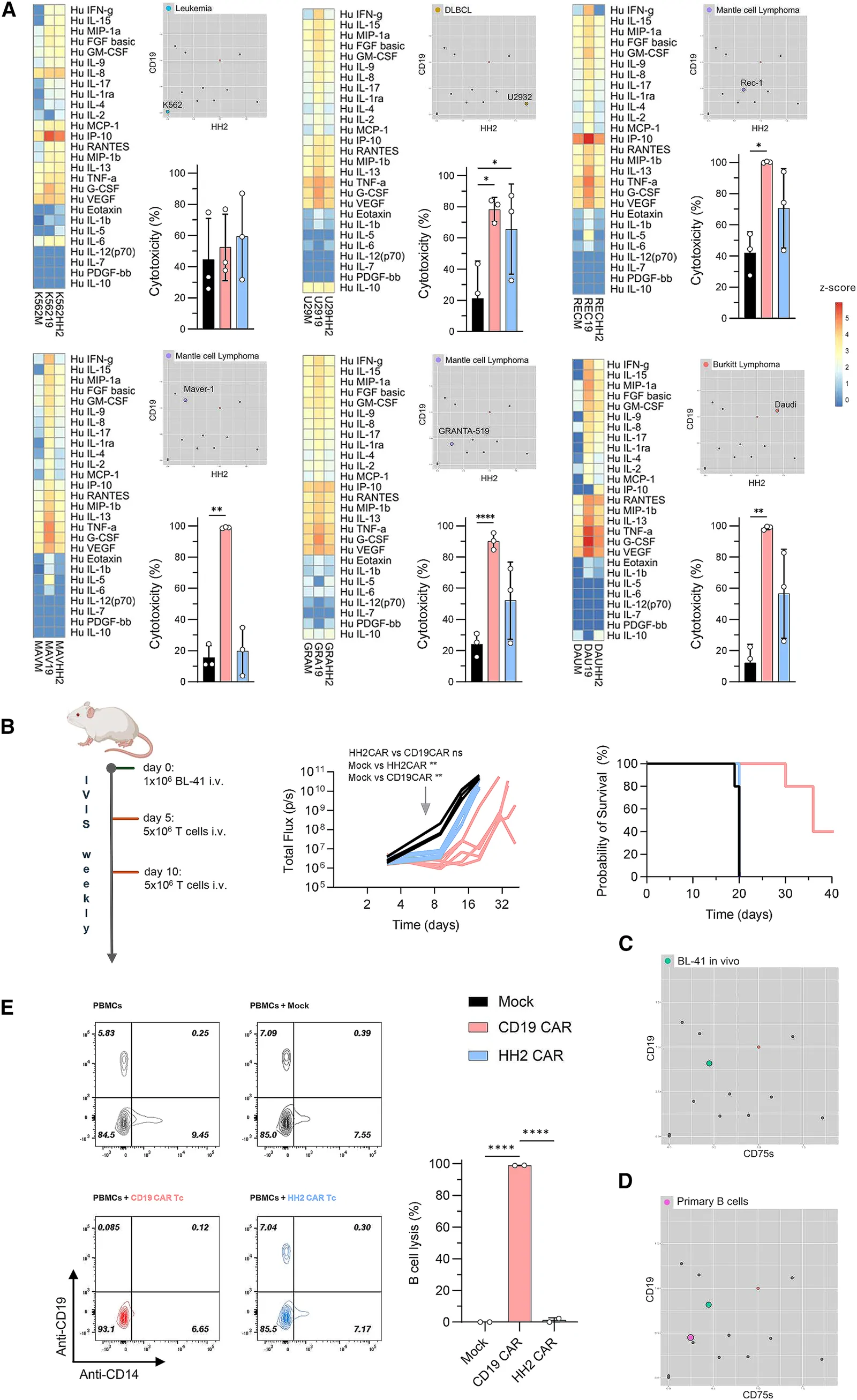
*In vivo* testing of HH2CAR in a BL-41 xenograft model (A) Each image represents a different cell line and is composed of (left) a cytokine profile and (right) scatterplot depicting HH2 fluorescence intensity (on the *x* axis) and CD19 fluorescence intensity relative to BL-41. *N* = 2 healthy donors; mean values are plotted. Cytotoxicity against the cell line highlighted after a 24-h co-culture is plotted as a histogram plot of the percentage of cell lysis with CAR T cells (CD19CAR in red and HH2CAR in blue) or with non-transduced T cells (mock, in black) (middle). *N* = 3 healthy donors; error bars represent SD. One-way ANOVA was used for statistics. Values (pg/mL) are plotted in a heatmap with a log10 scale; *N* = 1 healthy donor; mean values are plotted. (B) Left, *in vivo* experimental design for the BL-41 xenograft model. Top right, quantification of tumor load by IVIS during the experiment; the arrow indicates day 9 post-engraftment. One-way ANOVA was used to compare the groups at day 9. Bottom right, Kaplan-Meier curve of survival. (C) Scatterplot of CD19 and HH2 expression relative to BL-41 cells cultured *in vitro* (in red), highlighting BL-41 cells (in green) harvested from mice and their CD19 and HH2 expression pattern. *n* = 6 different mice; *N* = 3 independent experiments. Values plotted are the means of all individual values. (D) Scatterplot of CD19 and HH2 expression relative to BL-41 cells cultured *in vitro* (in red), highlighting healthy primary B cells (in purple) and their CD19 and HH2 expression pattern. *N* = 7 different donors; *n* = 3 independent experiments. Values plotted are the means of all individual values. (E) Primary B cell cytotoxicity of T cells either transduced with CD19CAR (in red) or HH2CAR (in blue), or non-transduced (mock, in black) against primary B cells from healthy and matching donors after 24 h co-culture. Left, representative contour plots of one donor. *N* = 2 healthy donors; the error bars represent SD; one-way ANOVA was used for statistics. *p* values are depicted as ∗∗∗∗*p* < 0.0001, ∗∗∗*p* < 0.001, ∗∗*p* < 0.01, and ∗*p* < 0.05, and *p* > 0.05 are non-significant (not shown).

### HH2-derived CAR design and assessment

We generated an scFv with a variable light (V_L_)-variable heavy (V_H_) orientation linked by a (G_4_S)_4_ linker based on the sequence identified from the HH2 hybridoma (Figure S1B). Notably, HH2 is an IgM antibody and, as expected, carries very few somatic hypermutations (Figure S1C). The second-generation CAR design was composed of a CD8α hinge and transmembrane (TM) domain and 4-1BB and CD3ζ domains (Figure 1H). HH2CAR T cells produced a wide range of cytokines once co-cultured with BL-41, as assessed with a 27-plex BioPlex assay, albeit at lower levels than CD19CAR T cells (Figure 1G). HH2CAR T cells were able to efficiently eliminate BL-41 cells (Figure 1I) and were selectively cytotoxic against CD75s-positive BL-41 cells but not against *ST6GAL1* KO BL-41 cells (Figure 1J). Surprisingly, HH2CAR T cells elicited no significant CD107a degranulation against BL-41 cells (Figure 1K). To verify that this was not restricted to BL-41, we tested different lymphoma models. We observed that the cytotoxic activity of the HH2CAR T cells correlated with the CD75s signal, but, importantly, none of these different target cell lines could elicit a strong cytokine release by the CAR T cells (Figure 2A). Collectively, these data reveal a paradox in the *in vitro* efficacy of HH2CAR T cells; while they demonstrate strong and specific cytotoxic activity, their cytokine production and degranulation potency remain comparatively weak.

### HH2CAR T cells are sensitive to CD75s antigen density

A mouse xenograft model was used as described (Figure 2B, left). Five days after engraftment of BL-41 cells, 2 intravenous (i.v.) injections of 5 × 10^6^ of the indicated T cells were performed, and tumor growth was quantified using bioluminescence (BLI) signals. As shown in this experiment, representative of 5 separate replicates, HH2CAR T cells provided an initial control of the tumor load detectable until day 9 (Figure 2B, middle), before tumor control was lost. Consequently, no survival benefit was observed with the HH2CAR T cells, which contrasted with the survival benefit provided by CD19CAR T cells (Figure 2B, right). To understand the cause of this low benefit with the HH2CAR T cells, we engrafted mice with BL-41 cells, which were harvested when the tumor load was high, and analyzed their CD75s and CD19 expression. Notably, *ex vivo* BL-41 cells exhibited markedly diminished CD75s density compared to plastic-cultured BL-41 cells (Figure 2C green versus coral-colored dot), suggesting that CD75s levels may significantly depend on specific microenvironmental cues. Interestingly, the same observation was made for CD19, although the reduction in intensity was much lower (Figure 2C). Since CD19CAR T cells can kill tumor cells with a low number of CD19 molecules at the surface,^26^ it is not surprising that the tumor could be controlled. To better understand the impact of dim CD75s antigen density on HH2CAR potency, we performed an experiment using primary B cells, which also show a low staining intensity with the HH2 mAb (Figure 2D). We tested the cytotoxic activity of HH2CAR T cells against those and observed that they could not kill primary B cells, whereas CD19CAR T cells did (Figure 2E). Taken together, these results showed that HH2CAR T cell functionality exhibits dependence on antigen density, precluding effective tumor control as a monovalent therapy.

### HH2CAR combined with CD19CAR

Given HH2CAR’s sensitivity to antigen density, we tested whether targeting CD75s could still be exploited in a combinatorial CAR architecture.^23^ Briefly, this combinatorial CAR design is composed of two CARs linked with a 2A-ribosome-skipping sequence, starting with HH2CAR in a first-generation design followed by CD19CAR with a 4-1BB intracellular domain (Figure 3A). This configuration, designated HH2/CD19 (4-1BB) CAR, was designed to implement a non-restrictive Boolean “AND” gate logic. Initially, two additional combinatorial designs were compared. All three constructs share the first CAR moiety—consisting of the HH2 scFv, a CD8-derived hinge and TM domain, and a CD3ζ signaling domain. In the additional two constructs, the second CAR molecule incorporates a CD28 costimulatory domain and is linked to the CD19 scFv by either a CD8 hinge and TM (HH2/CD19 (8-8-28) CAR) or a CD28 hinge and TM (HH2/CD19 (28-28-28) CAR), respectively (Figures 3A and S2A).

**Figure 3:**
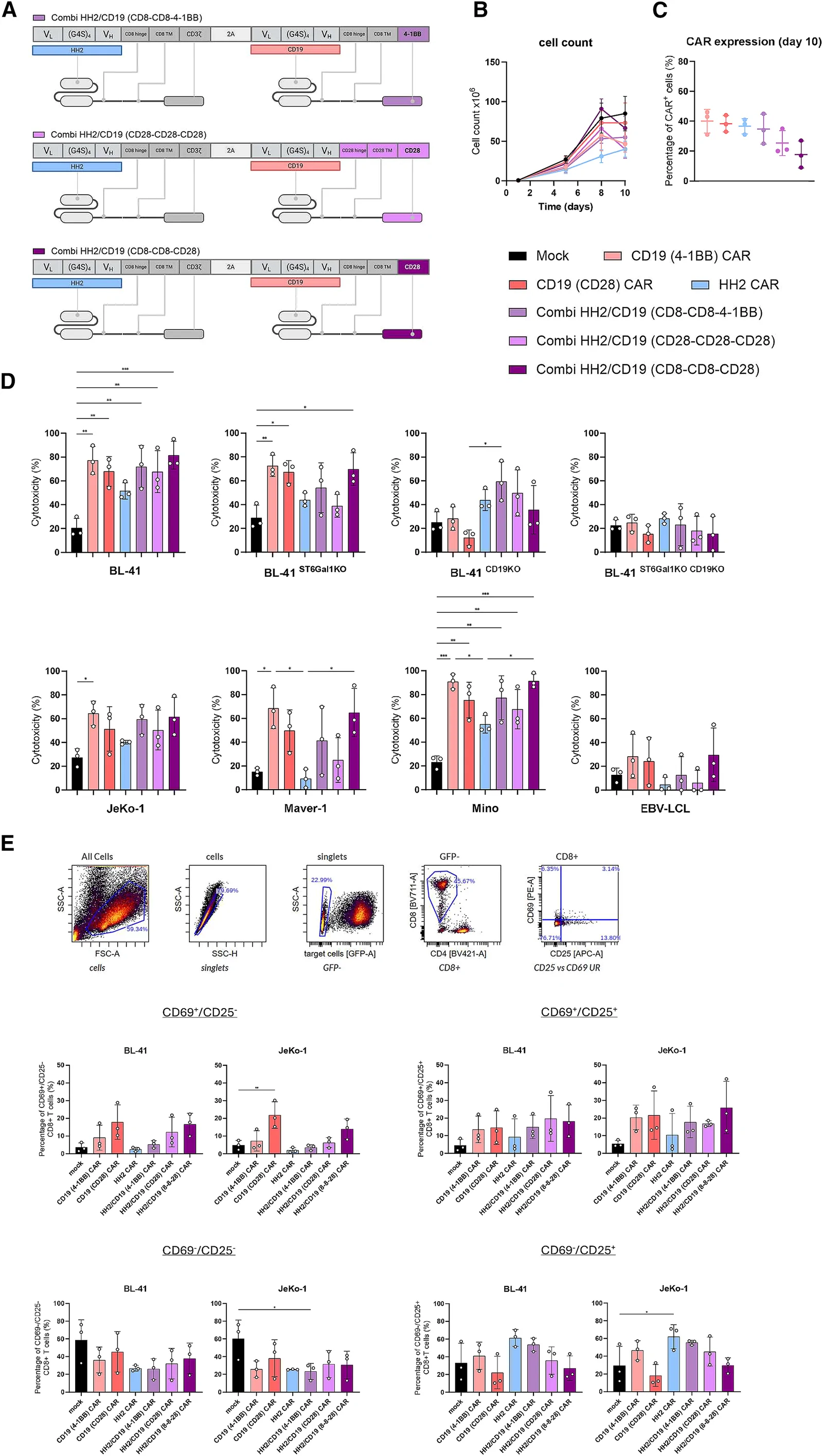
Comparison of different Combi HH2/CD19 designs (A) Graphical visualization of 3 different combinatorial designs. Top, DNA sequence schematic of HH2/CD19 (CD8^−^CD8-4-1BB) CombiCAR, consisting of an HH2 scFv (V_L_ region linked by (G_4_S)_4_ to V_H_ region), a CD8 hinge domain, a CD8 transmembrane (TM) domain, a CD3zeta, a 2A-ribosome-skipping sequence, a CD19 scFv (V_L_ region, (G_4_S)_4_ linker, and V_H_ region), a CD8 hinge, a CD8 TM, and a 4-1BB costimulatory domain. This translates to two CAR molecules, an HH2 CD3ζ-based moiety and a CD19 4-1BB-based moiety. Middle, HH2/CD19 (CD28^−^CD28^−^CD28) CombiCAR sequence and molecule schematic, highlighting differences in the CD19CAR moiety with a CD19 scFv connected to a CD28-derived hinge, CD28 TM, and CD28 costimulatory domain. Bottom, schematic of HH2/CD19 (CD8^−^CD8^−^CD28) CombiCAR DNA sequence and CAR proteins. (B) Time kinetics (in days) of T cell expansion (assessed as total cell count in millions) over a course of 10 days; *N* = 3. (C) CAR expression on day 10. FCM staining with anti-murine F(ab) (mFab) antibody and streptavidin-PE antibody. Depicted as percentage of CAR+ T cells; *N* = 3. (D) BLI-based cytotoxicity assay against BL41, BL41ST6Gal1KO, BL41CD19KO, BL41 ST6Gal1KO CD19KO, JeKo-1, MAVER-1, Mino, and EBV-LCL cells, evaluating the percentage of lysed target cells after 6 h of co-culture with CAR T cells. One-way ANOVA was used as the statistical test; the error bars represent SD; *N* = 3; *n* = 3. (E) CD25 and CD69 staining of CAR T cells after co-culture with BL-41 or Jeko-1 for 24 h. Top, gating strategy shown for staining mock T cells after co-culture (24 h) with BL-41 target cells. Exclusion of debris, selection of singlets, exclusion of GFP^+^ target cells (selection of GFP population), distinction of CD4 and CD8-positive CAR T cells, and plotting of CD69 versus CD25 signal to distinguish CD25 and CD69 double-negative, single-positive, or double-positive CD8^+^ T cells. Bottom, presentation of CD25/CD69 double-negative, CD69 single-positive, CD25 single-positive, or CD25/CD69 double-positive CD8^+^ CAR T cells after co-culture with BL-41 or JeKo-1. *N* = 3 healthy donors; *n* = 2 technical replicates; the error bars represent SD. Statistical test applied is one-way ANOVA, compared to mock T cells. *p* values are depicted as ∗∗∗∗*p* < 0.0001, ∗∗∗*p* < 0.001, ∗∗*p* < 0.01, and ∗*p* < 0.05, and *p* > 0.05 are non-significant (not shown).

By comparing these three combinatorial designs, a slight improvement in manufacturing could be observed for the 4-1BB-based HH2/CD19 CombiCAR over the CD28 incorporating CAR T cells (Figures 3B and 3C). Functional testing of the three CombiCARs in a cytotoxicity assay against JeKo-1, MAVER-1, Mino, BL-41, and relevant KO versions thereof revealed no significant differences in their ability to deplete these malignant B cell lines (Figure 3D). The Epstein-Barr virus (EBV)-lymphoblastoid cell line (LCL), which served as an immortalized surrogate for healthy B cells, did not get killed by the HH2 second-generation CAR, as well as the 4-1BB-based HH2/CD19 CombiCAR, and the HH2/CD19 (28-28-28) design, indicating potential sparing of healthy B cells by HH2-based CAR designs (Figure 3D).

To determine whether our combinatorial design affects T cell activation, we assessed the percentage of CD25^+^ and CD69^+^ T cells 24 h after co-culture with BL-41 or JeKo-1 target cell lines. CAR T cells tended to express higher levels of activation markers than mock T cells, with CD28-based CARs showing a trend toward a greater fraction of CD69^+^ CD8^+^ T cells, while 4-1BB-based designs appeared to favor CD25 upregulation instead. No significant differences were detected among the combinatorial designs (Figure 3E; Figure S2B). Given this, along with the advantages of 4-1BB-based designs reported in the literature, we selected the HH2/CD19 (4-1BB) CombiCAR for further *in vitro* and *in vivo* testing.

CAR-transduced T cells used for follow-up experiments had comparable surface expression at day 10 post-transduction, with no detrimental impact on *ex vivo* T cell expansion kinetics (Figures 4A and 4B). Functional assessment revealed that HH2/CD19CAR T cells exhibited enhanced CD107a degranulation compared to second-generation HH2CAR T cells (Figure 4D) but with levels still lower than those for CD19CAR T cells. Similarly, avidity profiling using the z-Movi platform demonstrated superior avidity of HH2/CD19CAR relative to HH2CAR, which exhibited poor baseline avidity (Figure 4E). Additionally, HH2/CD19CAR maintained a strict specificity toward the CD75s antigen, as demonstrated by restricted CD107a surface expression in co-culture assays with BL-41, either wild type (WT) or KO for *CD19*, *ST6GAL1*, or both (Figure 4D). Remarkably, HH2/CD19CAR T cells consistently demonstrated higher cytotoxic potency against the cell lines regardless of antigen density and displayed efficient cytotoxicity even against MAVER-1, which HH2CAR T cells failed to eliminate. Furthermore, *ST6GAL1*-deficient BL-41 cells, used to model an off-tumor/on-target scenario, exhibited reduced susceptibility to HH2/CD19CAR-mediated lysis compared to CD19CAR treatment. In contrast, the BL-41 *CD19*/*ST6GAL1* KO (double KO) abrogated cytotoxicity entirely (Figure 4C), indicating enhanced target discrimination. We further assessed CAR T cell activation by monitoring cytokine secretion using the BioPlex instrument. We observed that HH2/CD19CAR T cells produced higher amounts of cytokines when co-cultured with BL-41 cells than HH2CAR T cells, albeit less than CD19CAR T cells (Figure 4F). Lastly, we performed PBMC depletion assays, where PBMCs were co-cultured with autologous CAR T cells expressing the indicated CAR construct. HH2CAR T cells spared primary B cells, while CD19CAR and HH2/CD19CAR T cells both eliminated primary B cells. No significant toxicity against other cell subsets of PBMCs was observed for any of the CAR T cells (Figure 4G). Altogether, our data demonstrate that combining HH2 with CD19 targeting reinvigorated HH2CAR activity and that lower levels of CD75s are sufficient to trigger a full CAR response due to the efficient combination of the two targets.

**Figure 4:**
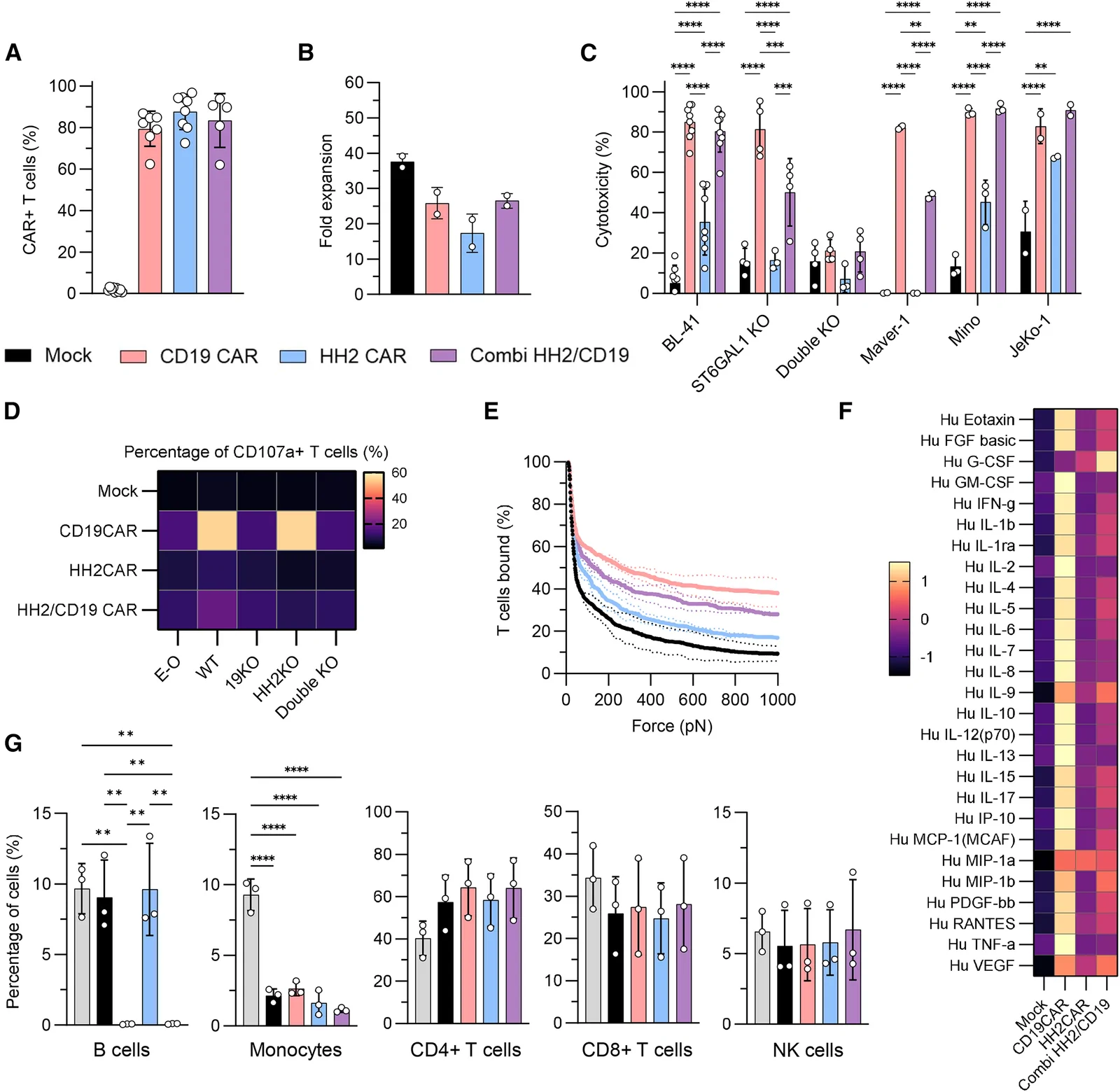
HH2CAR is potentiated by combinatorial design (A) Percentage of CAR^+^ T cells 10 days after transduction. Transduction efficiency of CARs in T cells measured by FCM using an anti-murine F(ab) (mFab) antibody at day 10 post-transduction. Each donor is represented by a dot, and SD is plotted as error bars. *N* = 7–9 healthy donors; one-way ANOVA was used for statistics. (B) Fold expansion of CAR T cells after 12 days of expansion. *N* = 2 healthy PBMC donors. (C) BLI-based cytotoxicity assay against indicated cell lines at an E:T ratio of 10:1, with T cells either transduced with CD19CAR (in red), HH2CAR (in blue), or Combi HH2/CD19CAR (in purple), or non-transduced (mock, in black) after 6 h of co-culture. *N* = 2–3 healthy donors; *n* = 2–3 independent experiments. Values plotted are means of each donor; the error bars represent SD; two-way ANOVA was used for statistics. (D) CD107a degranulation assay against BL-41 cells with T cells either transduced with CD19CAR (in red), HH2CAR (in blue), or non-transduced (mock, in black). Percentage of CD107a-positive cells is plotted, *N* = 4 healthy donors. (E) z-Movi functional avidity assay against BL-41 cells with T cells either transduced with CD19CAR (in red), HH2CAR (in blue), or Combi HH2/CD19CAR (in purple), or non-transduced (mock, in black). *N* = 1 healthy donor, *n* = 2 replicates. (F) BioPlex assay (27-plex) of cytokine production from supernatant of T cells either transduced with CD19CAR (in red), HH2CAR (in blue), or Combi HH2/CD19CAR (in purple), or non-transduced (mock) co-cultured with BL-41 cells for 24 h. *Z* score of cytokine production comparison is displayed. *N* = 2 healthy donors; each square represents mean values of the donors. (G) PBMC depletion assay with donor matching T cells either transduced with CD19CAR (in red), HH2CAR (in blue), or Combi HH2/CD19CAR (in purple), or non-transduced (mock) co-cultured with PBMCs for 24 h. Percentages of indicated subset of cells present after the co-culture are plotted. PBMCs in culture without T cells are in gray. *N* = 3 healthy donors; the error bars represent SD; one-way ANOVA was used for statistics. *p* values are depicted as ∗∗∗∗*p* < 0.0001, ∗∗∗*p* < 0.001, ∗∗*p* < 0.01, and ∗*p* < 0.05, and *p* > 0.05 are non-significant (not shown).

Besides antigen loss, the BL-41 xenograft model appeared too aggressive to benefit from HH2CAR T cell efficacy. Therefore, to assess the *in vivo* potency of the HH2/CD19CAR T cells, we selected the JeKo-1 cell line (a model of MCL), which, when cultured on plastic, exhibited a similar baseline level of CD75s to that of the BL-41 cells that grew in mice (Figures 1C and 2B). The JeKo-1 xenograft model was established as described (Figure 5A). Five days after engraftment of JeKo-1 cells, 2 i.v. injections of 5 × 10^6^ indicated T cells were administered, and tumor growth was quantified by BLI signal. HH2CAR T cells did not display strong control of tumor growth, while CD19CAR and HH2/CD19CAR appeared statistically as efficient (Figure 5A, right; Figure 5B). In this model, the HH2CAR T cells demonstrated a slight but significant benefit in survival (Figure 5C). CD19CAR and HH2/CD19CAR T cells both significantly improved survival (over 40 days for both groups). Consequently, these data demonstrate that the combinatorial design with CD19 targeting reinvigorated not only the *in vitro* activity of HH2CAR but also its *in vivo* potency.

**Figure 5:**
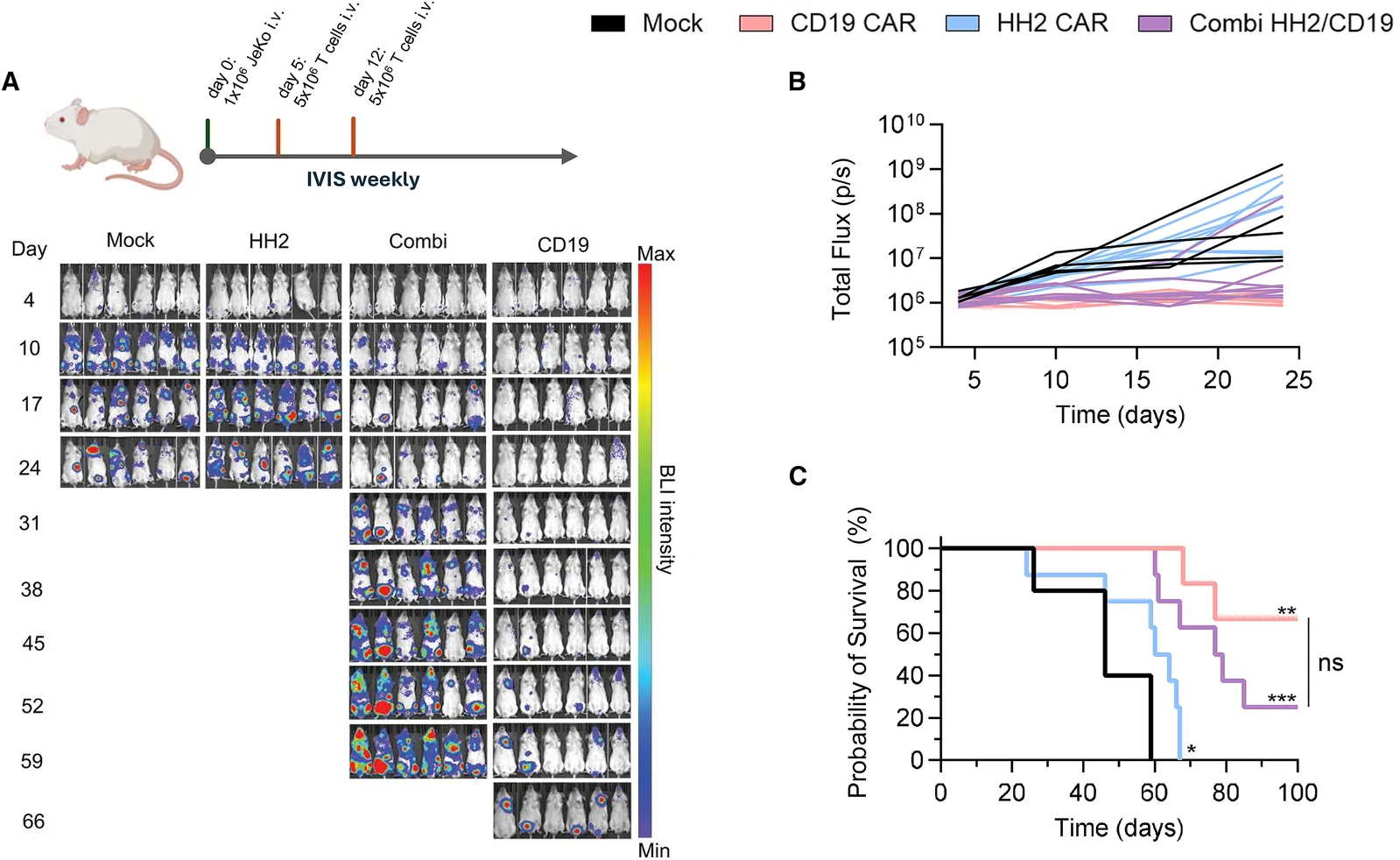
*In vivo* potency of Combi HH2/CD19 (A) Left, *in vivo* experimental design for the JeKo-1 xenograft model. Right, IVIS images of animals (*N* = 6) during the experiment. (B) Quantification of tumor load by IVIS during the experiment with *N* = 6. (C) Kaplan-Meier curve of survival probability with *N* = 6. *p* values are depicted as ∗∗∗∗*p* < 0.0001, ∗∗∗*p* < 0.001, ∗∗*p* < 0.01, and ∗*p* < 0.05, and *p* > 0.05 are non-significant (not shown).

## Discussion

Cancer aberrant glycosylation has been extensively described and shown to be functionally involved in cancer biology.^12^ Tumor-associated carbohydrate antigens (TACAs) have re-emerged as promising therapeutic molecular targets. The rationale for targeting TACAs focuses on minimizing off-tumor toxicity and mitigating therapy resistance due to antigen loss. However, generating mAb-derived scFvs specific to TACAs remains challenging, largely because immune responses to carbohydrates are T cell independent in nature, predominantly resulting in the production of IgM class antibodies and variable regions with little affinity maturation.^27^ This gets further complicated by the fact that purified glycans often lack sufficient immunogenicity to elicit specific antibody responses.^25^ Additionally, purified free glycans have a distinct structure compared to glycans covalently linked to a biological molecule, such as glycoproteins, in the appropriate cellular and molecular context.^28^ Similarly, efforts to generate mAbs using minimal protein scaffolds that present glycans in a native-like conformation risk eliciting antibodies that preferentially recognize the protein backbone rather than the glycan epitopes.^25^

In this study, we report a CAR derived from HH2, an mAb clone recognizing CD75s^29^^,^^30^ generated by ST6Gal1,^15^ first described in B cells and isolated 30 years ago.^10^ CD75s is recognized by the endogenous sialic acid-binding immunoglobulin-like lectin (Siglec) family, such as Siglec-2 (CD22) and Siglec-3 (CD33).^31^ Thus, CD75s is an attractive target because of its suspected role as an immune checkpoint, where aberrant glycosylation in the form of overexpression of sialylated residues, as observed in lymphomas, can be functionally involved in tumor immune escape.^32^ We first confirmed HH2 specificity for CD75s using neuraminidase treatment and *ST6GAL1* KO cell models, both of which resulted in complete abrogation of staining and loss of HH2CAR T cell activity. Thus, the HH2 scFv is efficient and functional and could be utilized as a functional second-generation CAR. However, although efficient cytotoxicity of HH2CAR was detected, low cytokine production was observed. Similarly, HH2CAR T cells exhibited only transient tumor control in BL-41 and JeKo-1 xenograft models, resulting in delayed but not durable suppression of tumor growth. Interestingly, we observed that BL-41 cells harvested after *in vivo* expansion displayed 2-fold lower expression levels of CD75s staining compared to the same cells cultured *in vitro*, yet conserved a similar percentage of expressing cells. Additionally, a loss of CD19 expression was observed, although less markedly. The reduced expression of CD75s observed in BL-41 cells *ex vivo* was comparable to the levels we observed in resting B cells isolated from PBMCs. Yet, primary B cells were spared by HH2CAR T cells in PBMC depletion assays, which demonstrated the sensitivity of HH2CAR to antigen density on target cells. Previous reports have demonstrated that factors like culture confluency and the differentiation state of cancer cells greatly impact the levels of surface expression of α2,6-sialylated molecules.^33^^,^^34^ Thus, considering the high expression of CD75s reported for lymphoma and other carcinomas^35^ (gastric cancer,^18^^,^^19^ breast cancer,^20^ and colorectal cancer^21^), CD75s appears as an attractive target, and we therefore aimed to further develop HH2CAR.

It is tempting to speculate that monovalent targeting of CD75s via an scFv may fail to generate a sufficiently robust activation signal in T cells upon antigen recognition because the HH2 mAb is of the IgM isotype. Indeed, CAR constructs are mainly derived from IgG, which are more mature and normally have higher affinity and specificity for their target. IgM mAbs on the other hand are usually of low functional avidity and are thus more sensitive to antigen density.^36^

To increase the CAR affinity of the engineered T cells, we investigated whether a combinatorial CAR design with CD19 (HH2/CD19CAR)^23^ could circumvent the low avidity and rescue the potency while further restricting CD19 targeting to avoid on-target/off-tumor toxicities associated with CD19.^8^ Therefore, we first compared a 4-1BB versus a CD28 signaling domain-based design and found no significant difference between the 3 combinatorial designs (Figure 3). Based on these tendencies, hinting at improved manufacturing of the 4-1BB-based combinatorial CAR, as well as a good balance between potency and specificity, we decided to continue with the HH2/CD19 (4-1BB) CombiCAR design. Other authors have reported an advantage of 4-1BB-based designs, claiming that CD28^−^CD3ζ-based CAR T cells differentiate more into short-lived effector states, whereas 4-1BB-CD3ζ-based designs preferentially give rise to durable memory offspring,^37^ further supporting our decision. Indeed, HH2/CD19 (4-1BB) CAR T cells displayed increased functional avidity toward BL-41 cells compared to HH2CAR T-cells. Additionally, with the HH2/CD19CAR T cells, we observed increased cytokine production compared to that for HH2CAR, although at levels lower than those for the CD19CAR T cells. This hints at improved potency over the HH2CAR, but at the same time, it indicates a potentially lower risk for cytokine-associated side effects reported for CD19CAR.^38^ We also observed a tendency for improved T cell expansion compared to HH2CAR. As the BL-41 xenograft model was an aggressive model with a short timeline for assessing therapeutic efficiency, and since this cell line exhibited a downregulation of CD75s upon *in vivo* culture, we selected JeKo-1 cells that exhibited a similar *in vitro* antigen density to *in*-*vivo*-grown BL-41 cells and to primary B cells. HH2/CD19CAR T cells conferred a significant survival benefit *in vivo* with the JeKo-1 xenograft model, which correlates with the observed reinvigoration *in vitro*, although the survival benefit was lower than that observed in mice treated with CD19CAR T cells. This observation, along with the primary B cell cytotoxicity of HH2/CD19CAR T cells, demonstrates that HH2/CD19CAR reduced CAR dependency on antigen density and can efficiently target cells with reduced CD75s cell surface density. Although in our cytotoxicity assay against EBV-LCLs, the results indicate that these healthy-like B cells, which express less CD75s, in fact get killed less than their malignant counterpart. Therefore, HH2/CD19CAR T cells might induce B cell aplasia, which is clinically manageable,^39^ but could further restrict CD19-related toxicities as the activation of HH2/CD19CAR T cells is primarily directed by CD75s recognition. The improved cytokine production potency observed for HH2/CD19CAR T cells as well as the increased HH2/CD19CAR T cell functional avidity suggest that the CD19-targeting moiety contributes to the functional avidity and potency. Similar results were reported with CD19^−^ and BCMA combinatorial CARs with the addition of a chimeric costimulatory receptor (CCR), among which one of the designs is close to our HH2/CD19CAR.^24^ Collectively, our data demonstrate that a combinatorial approach not only allows for multiplexed targeting but also enhances functional avidity and cytokine production.

In conclusion, this study provides evidence that low-affinity mAb-derived CARs can be reinvigorated by combinatorial design approaches and highlights distinct functional consequences of combinatorial design depending on the scFv used. Additionally, the study proposes a strategy that could improve the safety of CD19CAR T therapy for lymphomas. The findings warrant further investigation, including the screening of different combinatorial designs to tailor the design to the HH2-derived scFv and target, as well as the exploration of other combinations of recognition domains.

### Limitations of the study

This proof-of-concept study aimed to test whether a low-affinity glycan-targeting antibody could be repurposed by pairing it with a lineage-marker antibody to restrict tumor specificity. Although we demonstrate that this combination yields a functional construct, further evaluation using alternative companion scFvs would provide insights into the universality of this approach. In addition, subtle differences in efficacy were difficult to capture *in vitro* due to the high potency of the CD19CAR. Nevertheless, the combinatorial design offers the advantage of restricting CD19-dependent activation to double-positive targets. However, this strategy remains susceptible to antigen loss, which is a common mechanism of relapse in CD19-directed therapies. In contrast, sialylation appears to confer a survival advantage to tumor cells; therefore, aberrant glycosylation is likely to be retained even if lineage-marker expression changes. Another limitation of this study is that the contribution of the tumor microenvironment (TME) could not be assessed. Evaluating human CAR T cells *in vivo* requires the use of immunocompromised xenograft hosts to prevent rejection of the infused T cells by the murine immune system. This necessary feature of the model precludes assessment of interactions with other components of the host immune system. Similarly, to our knowledge, current *in vitro* assays do not adequately recapitulate the contributions or suppressive activities of other TME-resident populations, such as natural killer (NK) cells, myeloid-derived suppressor cells (MDSCs), and regulatory T cells (Tregs). As a result, the influence of the TME on HH2/CD19 CombiCAR efficacy could not be addressed in the present study and remains an important avenue for future investigation.

## Resource availability

### Lead contact

Further information and requests for resources and reagents should be directed to and will be fulfilled by the lead contact, Sébastien Wälchli (sebastw@ous-hf.no).

### Materials availability

All unique reagents generated in this study are available from the lead contact upon request.

### Data and code availability

• Raw data have been deposited at Mendeley repository and are publicly available as of the date of publication: https://data.mendeley.com/preview/z84vwn2bwt?a=42e3d265-bbbb-4e77-85f8-05f8cd298cb2.
• The published article does not report the custom computer code.
• Any additional information required to reanalyze the data reported in this study is available from the lead contact upon request.

## Acknowledgments

We thank Wafa Kefi, Emilie Einersten, Karoline Strømmen Martinsen, and Dr. Gwenann Cadiou (OUH) for PBMC preparation and cell sorting. We are grateful to the Radiumhospitalets legater and the Jonathans Minnefond for their support. We thank Dr. Idun Dale Rein, Monica Bostad, and Heidi Ødegård Noto from the Flow Cytometry Core Facility and the Department of Comparative Medicine at Oslo University Hospital (OUH). C.F. was a PhD fellow of the 10.13039/501100011755National Center for Research and Development within the POLNOR program NOR/POLNOR/ALTERCAR/0056/2019 and was then a postdoc from the Norwegian Health Authority West, Helse Vest (MyCAR). S.W. received funding from the 10.13039/100008730Norwegian Cancer Society (IIDEA, no. 223171), the 10.13039/501100016242Norwegian Childhood Cancer Society (no. 230004 [PERCAP] and no. 240005 [PEDHEMA]), the 10.13039/501100005416Research Council of Norway (nos. 337468, 351914, and 351915), and the Norwegian Health Authority South-East (nos. 2024080, 2022009, and 2016006). E.M.I. received funding from the 10.13039/501100005416Research Council of Norway through KSP-2021 (CellFit, no. 326811). H.O.D. and C.G. were supported by the individual contracts 2022.00943.CEECIND (https://doi.org/10.54499/2022.00943.CEECIND/CP1735/CT0017) and 2022.04678.CEECIND, both from the 10.13039/501100019370Portuguese Foundation for Science and Technology. C.A.R. acknowledges FCT (PTDC/MEC-ONC/0491/2021). E.M. acknowledges the support provided by the 10.13039/501100005416Research Council of Norway (no. 326300), the 10.13039/100008730Norwegian Cancer Society (IIDEA, no. 223171), the 10.13039/501100016242Norwegian Childhood Cancer Society (no. 240005 [PEDHEMA]), and the Norwegian Health Authority West (MyCAR).

## Author contributions

Conceptualization, S.W., E.M.I., E.B.S., and J.H.M.; methodology, S.W., E.M.I., P.G., and J.H.M.; investigation, C.F., H.K., E.S., R.A., P.D., H.O.D., S.J., C.G., C.d.M., H.T.Z.H., and R.D.; software, C.F., H.K., E.S., R.A., P.D., H.O.D., S.J., C.G., C.d.M., H.T.Z.H., and R.D.; visualization, C.F., H.K., and E.S.; writing, H.K., C.F., and S.W.; editing, E.S.; review, all authors; and supervision, S.W., E.M.I., P.G., E.M., C.A.R., J.H.M., and E.B.S.

## Declaration of interests

The authors declare no competing interests.

## STAR★Methods

### Key resources table

**Table:** 

REAGENT or RESOURCE	SOURCE	IDENTIFIER
**Antibodies**		

anti-CD19-BV510 (HIB19)	BioLegend	Cat#302242; RRID: AB_2561668
anti-immunoglobulin (Ig)λ–Pacific Blue (MHL-38),	BioLegend	Cat#316606; RRID: AB_493625
anti-CD3 BV785 (OKT3)	BioLegend	Cat#317329; RRID: AB_11219196
anti-CD20–APC–H7 (L27)	BD Biosciences	Cat#340941; RRID: AB_400553
anti-Igκ–PerCPCy5.5 (G20-193)	BD Biosciences	Cat#561330; RRID: AB_10611735
anti-CD75s-Alexa 488 (HH2)	David Warren, Radiumhospitalet OUS, Oslo	N/A
anti-CD19-PE (HIB19),	BD Biosciences	Cat#555413; RRID: AB_395813
HH2 hybridoma	David Warren, Radiumhospitalet OUS, Oslo	N/A
anti-mouse IgG-APC	BioLegend	Cat#405308; RRID: AB_315011
anti-CD3-BV605(SK7)	BD Biosciences	Cat#563219; RRID: AB_2714001
anti-CD56-FITC (NCAM16.2)	BD Biosciences	Cat#345811; RRID: AB_2868832
anti-CD14-APC-Cy7 (61D3)	Thermo Fisher Scientific	Cat#11-0149-42; RRID: AB_10597597
anti-mouse Fab	Jackson ImmunoResearch	Cat#115-066-072; RRID: AB_2338583
Streptavidin-PE	BD Biosciences	Cat#554061; RRID: AB_10053328
anti-CD4-BV421 (L200)	BD Biosciences	Cat#562842; RRID: AB_2737832
anti-CD8-BV711 (RPA-T8)	BD Biosciences	Cat#563677; RRID: AB_2744463
anti-CD25-APC (BC96)	BD Biosciences	Cat#567316; RRID: AB_2916552
anti-CD69-PE (FN50)	BD Pharmingen	Cat#555531; RRID: AB_395916
anti-CD107a-PE-Cy5 (H4A3)	BD Biosciences	Cat#555802; RRID: AB_396136
anti-CD3 (OKT3)	Thermo Fisher Scientific	Cat#16-0037-85; RRID: AB_468855
anti-CD28 (CD28.6)	Thermo Fisher Scientific	Cat#16-0288-85; RRID: AB_468925

**Bacterial and virus strains**		

NEB® 5-alpha Competent E. coli (High Efficiency) | DH5α	New England Biolabs	Cat#C2987H

**Biological samples**		

Human peripheral blood mononuclear cells (PBMCs)	Healthy donors	(OUS REK ID 2019-121)

**Chemicals, peptides, and recombinant proteins**		

Recombinant human IL-2 (Proleukin)	Clinigen	N/A
TritonTM X-100	Sigma-Aldrich	X100
Gentamycin	Gibco	Cat#15750-037
Cell Trace Violet	Thermo Fisher Scientific	Cat#C34557
Propidium Iodide	Thermo Fisher Scientific	Cat#R37169
Dimethyl sulfoxide (DMSO)	Sigma Aldrich	Cat#D5879
GolgiStop	BD Biosciences	Cat#554724
GolgiPlug	BD Biosciences	Cat#555029
D-Luciferin potassium salt	Revvity	Cat#122799
Retronectin	Takara Bio.	Cat#T100B
Neuraminidase	Roche	Cat#11585886001
Puromycin	Gibco	Cat#A1113802
BD Fc Block	BD Biosciences	Cat#564220
Deoxyribonuclease (DNAse) I from bovine pancreas	Sigma Aldrich	Cat#DN25
DAPI	Sigma Aldrich	Cat#D9542
CTS™ Immune Cell Serum Replacement (SR)	Thermo Fisher Scientific	Cat#A2596101
X-VIVO® 15 Serum-free Hematopoietic Cell Medium	Lonza	Cat#02-053Q
RPMI 1640	WVR	Cat#392-0426
Fetal bovine serum (FBS)	Gibco	Cat#10500-064
Dynabeads® ClinExVivo™	Thermo Fisher Scientific	

**Critical commercial assays**		

Bio-Plex Pro Human Cytokine 27-plex assay	Bio-rad	Cat#M500KCAF0Y
Normal Rabbit Serum	Dako	Cat#X090210-8
Paraformaldehyde (PFA)	Thermo Fisher Scientific	Cat#AAJ61899AK

**Experimental models: Cell lines**		

Maver-1	DMSZ	ACC 717
Mino	DMSZ	ACC 687
JeKo-1	ATCC	CRL-3006
Rec-1	DMSZ	ACC 584
K-562	ATCC	CCL-243
U2932	DMSZ	ACC 633
Daudi	J. Myklebust (Oslo University Hospital)	Köksal et al.^40^
GRANTA-519	J. Myklebust (Oslo University Hospital)	Köksal et al.^40^
BL-41	DMSZ	ACC 160
Sc-1	DMSZ	ACC 558
HEK-P (Phoenix-AMPHO)	LCG Genomics GMBH	ATCC-CRL-3213
Jurkat76 (J76)	M. Heemskerk (Leiden University Medical Center, The Netherlands)	Heemskerk et al.^41^
Caco-2	ATCC	HTB-37
B95-8	ATCC	CRL-1612

**Experimental models: Organisms/strains**		

NOD.Cg-Prkdc^scid^ Il2rg^tm1Wjl^/SzJ (NSG) mice	The Jackson Laboratory	SCANBUR AS

**Oligonucleotides**		

CD19 sgRNA: CTGTGCTGCAGTGCCTCAAGGG	Eurofins	N/A
*ST6GAL1* sgRNA: TGTATCCTCAAGCAGCACCC	Celso Reis	Duarte et al.^42^

**Recombinant DNA**		

Plasmid: pMP71-G		Wälchli et al.^43^
Plasmid: pRDF	Martin Pule University College London, London, United Kingdom	Cosset et al.^44^
Plasmid: pPeQ-PaM3-env	Martin Pule University College London, London, United Kingdom	Persons et al.^45^
Plasmid: pSIRV-NFAT-eGFP	Peter Steinberger	https://n2t.net/addgene:118031; RRID: Addgene_118031
Plasmid: pDEST51-EGFP		N/A
Plasmid: MSCV_Cas9_puro	Christopher Vakoc	https://n2t.net/addgene:65655; RRID: Addgene_65655
Plasmid: GFP-Luc	Rainer Löw	Loew et al.^46^

**Software and algorithms**		

FlowJo v10.10.0	FlowJo, LLC	https://www.flowjo.com/
BioRender	BioRender	https://www.biorender.com/
GraphPad Prism v10.6.1	GraphPad software	https://www.graphpad.com
Cytobank Community	Beckman Coulter	https://community.cytobank.org/
Cytobank Premium	Beckman Coulter	https://premium.cytobank.org/
IVIS®-200 imaging system	Perkin Elmer	https://www.perkinelmer.com/
R 4.2.0	R Core Team	https://www.r-project.org/
RStudio 2022.07.1 + 554	Posit	https://posit.co/products/open-source/rstudio/
AxioVision Release 4.8	ZEISS Microscopy	https://www.zeiss.com/microscopy/us/service-support/support/discontinued-products.html

### Experimental model and study participant details

#### Patient material and cell lines

Cryopreserved cells from malignant lymph nodes obtained at time of diagnosis were from follicular lymphoma (FL, *N* = 5, age 49–67, 2 female, 2 male, 1 unknown sex), diffuse large B cell lymphoma (DLBCL, *N* = 17, age 46–79, 4 female, 13 male), mantle cell lymphoma (MCL, *N* = 10, age 63–85, 4 female, 6 male), chronic lymphocytic leukemia (CLL, *N* = 8, age 54–74) and from healthy donor tonsils (*N* = 8, anonymized) and PBMCs (*N* = 2, anonymized). The human cell lines BL-41, GRANTA-519, DAUDI, K562, MAVER-1, MINO, REC-1, SC-1 and U2932 were acquired from Deutsche Sammlung von Mikroorganismen und Zellkulturen (DMSZ, Germany) and SU-DHL-4 line was a kind gift from L. M. Staudt (National Cancer Institute, National Institutes of Health). Jurkat-76 (J76) cell line was a kind gift from M. Heemskerk (Leiden University Medical Center, The Netherlands). Hek-Phoenix-AMPHO and JeKO-1 cells were acquired from American Type Culture Collection (ATCC, USA). Epstein-Barr virus-transformed lymphoblastoid cell lines (EBV-LCL) were prepared in house by immortalization of healthy human PBMCs with EBV using supernatants from the Mamorset cell line B95-8. All cell lines were regularly monitored for the presence of mycoplasma (Venor GeM; Minerva Biolabs, Berlin, Germany). The cells were maintained in Roswell Park Memorial Institute (RPMI) 1640 medium supplemented with 10% fetal bovine serum (FBS) (both from PAA Laboratories, Cölbe, Germany) and 0.05 mg/mL gentamycin (Garamycin; Schering-Plough Europe, Brussels, Belgium).

#### Mouse xenograft studies

NOD.Cg-Prkdc^scid^ Il2rg^tm1Wjl^/SzJ (NSG) mice were bred in-house and maintained in pathogen-free conditions under an approved institutional animal care protocol. 6- to 10-week-old female NSG mice were injected intravenously with 10^6^ luciferase-expressing BL-41 cells. After 3 days, tumor load was assessed with IVIS imaging and animals were randomized. The following day, without expansion 5 × 10^6^ CAR T-cells were injected intravenously. Tumor growth was monitored by weekly IVIS imaging. Plotting and statistical analysis were performed using GraphPad Prism software (La Jolla, CA, USA).

#### Ethical approval

All investigations were carried out according to the 2013 revision of the Declaration of Helsinki. PBMCs were obtained from healthy donors according to the Regional Committee for Medical Research Ethics South-East (OUS REK IDs 2013–624 and 2019–121). The animal study protocol was approved by the Institutional Review Board (or Ethics Committee) of Norwegian Food Safety Authority (OUS FOTS IDs 9597, 26731 and 11118).

### Method details

#### Immunophenotyping by flow cytometry

Patient specimens were stained with anti-CD19-BV510 (HIB19), anti-immunoglobulin (Ig)λ–Pacific Blue (MHL-38), and anti-CD3 BV785 (OKT3) (all from BioLegend, San Diego, CA), and anti-CD20–APC–H7 (L27) and anti-Igκ–PerCPCy5.5 (G20-193) (all from BD Biosciences, San Diego, CA), in addition to anti-CD75s-Alexa 488 (HH2) from in-house collection.

The cell lines were stained with CD19-PE (HIB19), in-house produced anti-HH2 followed by anti-mouse IgG-APC (Biolegend). Peripheral blood mononuclear cells (PBMCs) from healthy donors were additionally stained with CD3-BV605, CD56-FITC, CD14-APC-Cy7. CAR expression was measured by anti-mouse Fab (Jackson ImmunoResearch, West Grove, PA, USA) and streptavidin-PE (BD Biosciences). Flow cytometry was performed on BD FACSCanto II (BD Biosciences) and analyses were either performed with FlowJo software (BD Biosciences) or online Cytobank software ((Beckman Coulter).

#### Immunofluorescence

Cells were grown onto 13 mm glass slides and fixed with 4% paraformaldehyde for 5 min at RT. Slides were permeabilized with 0.5% Triton X-100 (Sigma-Aldrich) for 10 min at 4°C. Cells were blocked with 1:5 dilution of normal rabbit serum (Dako) in 10% BSA for 30 min at RT, and incubated with the HH2 antibody (hybridoma supernatant, 1:5) overnight (ON) at 4°C. Cells were labeled with 1:500 Alexa Fluor 594-conjugated IgM-specific secondary antibody (Invitrogen) for 45 min at RT and underwent nuclear staining with DAPI (Sigma-Aldrich). Images were acquired with a Zeiss Axio cam MRm and the AxioVision Rel. 4.8 software (Zeiss).

#### Generation of knockout cell lines

The CRISPR-Cas9 system was utilized to generate *CD19* and *ST6GAL1* knockout (KO) BL-41 cell line. GFP-Luc expressing BL-41 cells were further modified to express Cas9-Puro construct. Transduced BL-41 cells were selected by puromycin (2,5λ, Puromycin Dihydrochloride, Thermo Fischer Scientific). Afterward, 2 × 10^6^ BL-41 cells were electroporated with roughly 400pmol of commercial sgRNAs (CD19 sgRNA, *CTGTGCTGCAGTGCCTCAAGGG*; *ST6GAL1* sgRNA,^47^ TGTATCCTCAAGCAGCACCC). Around day 5, electroporated cells were phenotyped as described above to monitor the expression of the target. Cells were sorted by SH800S Cell Sorter system (Sony Biotechnology Inc., California, USA) to increase the percentage of KO cells. Sorted cells were then subjected to clonal expansion in 96-well plate at approximately 1 cell/well. In two weeks, a portion of cells from different wells were monitored for the KO of the target and a negative clone was selected for the following experiments.

#### DNA constructs and retrovirus preparation

The complete method is published elsewhere.^40^ Briefly, total messenger RNA (mRNA) was isolated from HH2 hybridoma.^21^ To identify the variable heavy and light chain fragments 5′ rapid amplification of complementary DNA ends (5′-RACE) was performed. The same second-generation CAR (4-1BB) design in our previous publication was used.^20^ CD19CAR (fmc63 clone) single chain variable fragment (scFv) was a kind gift from Martin Pule University College London, London, United Kingdom. A retroviral construct of firefly luciferase-GFP was a kind gift from Rainer Löw, BioNTech IMFS, Idar-Oberstein, Germany. Cell lines were transduced as described before^49^ and sorted according to GFP expression.

#### *In vitro* expansion of human T cells

Human PBMCs are extracted from healthy donors from the blood bank. As these donors are anonymized, no data on sex or age are available. PBMCs are isolated by a protocol adapted from T cell production under GMP conditions as previously described.^40^ In brief, PBMCs were separated from blood by density gradient and cultured in the presence of Dynabeads (Dynabeads® ClinExVivo CD3/CD28, Thermo Fisher, Oslo, Norway) in X-VIVO 15 (Lonza, Basel, Switzerland) supplemented with 100U/mL recombinant human IL-2 (Clinigen) and 5% CTS serum replacement (Thermo Fischer Scientific). Fresh culture media was added every other day for the duration of expansion (10–11 days). Cell numbers and viability were evaluated by Countess II Automated Cell Counter (Thermo Fischer Scientific).

#### Retroviral transduction of human T cells

Retroviral supernatants were collected as previously described.^40^ PBMCs were isolated from healthy donors as described above. A 24-well plate coated with anti-CD3 (1 μg/mL, OKT3 clone, Thermo Fisher, Norway) and -CD28 (1 μg/mL, CD28.6 clone, Thermo Fisher, Norway) and isolated PBMCs were transferred at 1 × 10^6^ cells/well in X-VIVO 15 complete medium. Cells were then left for 3 days at 37°C in 5% CO2. Activated T cells were transferred to a retronectin (50 μg/mL, Takara Bio. Inc., Shiga, Japan) coated 24-well plate (Nunc A/S, Roskilde, Denmark). T cells were then mixed with retroviral supernatants and spinoculated at 32°C at 750 g for 60 minutes. The spinoculation was repeated once more the following day with fresh medium and retroviral supernatant. T cells were then washed twice with complete X-VIVO 15 culture medium and cultured in the same medium for 3–4 days. Afterward, transduction efficacy was evaluated by flow cytometry and if satisfactory transduced cells were expanded with anti-CD3/CD28 Dynabeads as described above.

#### Neuraminidase treatment

BL-41 cells were prepared at a concentration of 1 × 10^7^ cells/mL in PBS. Neuraminidase (Sialidase, Sigma-Aldrich) was introduced at a final concentration of 1U/mL and cells were cultured for 30 min at 37°C in 5% CO2. Afterward, cells were washed three times with complete RPMI-1640. At each time point, cells were fixed with 2% paraformaldehyde for 60 min. Sialic acid expression was confirmed by flow cytometry following the phenotyping protocol by anti-HH2 as described above.

#### Functional assay

T cells were co-cultured with target cells at an effector to target ratio of 1:2 for 6 h at 37°C in 5% CO2. Prior CD107a-PE-Cy5 antibody (BD Biosciences, USA) was added to the cells together with Golgi Plug and Golgi Stop (BD Biosciences) prior to incubation. Cells were then stained with anti-CD3-BV605 (BD Biosciences) to specifically gate on T cells. Before and after staining, cells were washed with flow buffer (FB, phosphate-buffered saline (PBS) with 2% FBS). Cells were acquired on a BD FACSCanto II flow cytometer and the data analyzed using FlowJo software. Plotting and statistical analysis were performed using GraphPad Prism software (La Jolla, CA, USA).

#### Cytokine quantification

CAR-T cells and target cells were resuspended in plain X-VIVO-15 media and plated at an E:T ratio of 1:2 in a 96-well plate in duplicates. After 24 h, the cells were spun down, and the supernatant was transferred into another plate and stored at −80°C. Upon thawing, supernatants were diluted 1:3 in plain X-VIVO 15. Cytokines were quantified using the Bio-Plex Pro Human Cytokine 27-plex assay on a Luminex 200 system (Bio-rad). Plotting and statistical analysis were performed using GraphPad Prism software (La Jolla, CA, USA).

#### Bioluminescence-based cytotoxicity assay

The killing assay was performed as previously described.^40^ Briefly, luciferase-expressing target cells were prepared as 3 × 10^5^ cells/ml in the presence of 75 μg/mL XenoLight D-Luciferin potassium salt (PerkinElmer, Waltham, MA) and transferred to a 96-well white plates in triplicate. Subsequently, T cells were added at the indicated effector-to-target (E:T) ratios and incubated in a 5% CO_2_ incubator at 37°C. Bioluminescence (BLI) was measured with a luminometer (VICTOR Multilabel Plate Reader; PerkinElmer) as relative light units (RLU). Replicate wells were averaged, and lysis percentage was calculated using the following equation: percentage specific killing = 100 × (spontaneous cell death RLU − sample RLU)/(spontaneous death RLU − maximal killing RLU). Plotting and statistical analysis were performed using GraphPad Prism software (La Jolla, CA, USA).

#### CD25/CD69 activation marker assay

To test for upregulation of the activation markers CD25 and CD69, CAR-T cells were co-cultured with target cells (BL-41 and JeKo-1) in an effector to target ratio of 1:2. 24h after co-culture, cells were washed with flow buffer, blocked with Fc Block (BD) for 15 min at RT, before being stained with CD4-BV421, CD8-BV711, CD25-APC, and CD69-PE. Samples were acquired on a Cytek Aurora 5-laser Instrument in conventional mode. Gating and analysis were performed on the web-based Cytobank community platform (Beckman Coulter). Plotting and statistical analysis were made using GraphPad Prism software 10 (La Jolla, CA, USA).

### Quantification and statistical analysis

Statistical details on each experiment can be found in the figure legend. Briefly, N is the number of biological replicates (independent experiments) and n represents the number of technical replicates. Statistical tests used are described in the figure legend with tests performed being one-way or two-way ANOVA using the GraphPad Prism 10 software. *p*-values are indicated in each figure and are depicted as ∗∗∗∗*p* < 0.0001, ∗∗∗*p* < 0.001, ∗∗*p* < 0.01, ∗*p* < 0.05, and *p* > 0.05 are non-significant (with non-significant *p*-values not specifically depicted in figures).

## Supplemental information

Document S1. Figures S1 and S2

Document S2. Article plus supplemental information
