# Supplementary material for: Reviving low-affinity glycan-targeting CAR with combinatorial design
Source: Cell Rep Med. 2026 Oct 8;7(10):103108. doi: 10.1016/j.xcrm.2026.103108 (PMC13710698; doi:10.1016/j.xcrm.2026.103108)
Supplement: Supplementary file 1 [file mmc1.pdf]

Cell Reports Medicine, Volume *7*

Supplemental information

Reviving low-affinity glycan-targeting CAR with combinatorial design

Christopher Forcados, Hakan Köksal, Eveline Scherer, Rafaela Abrantes, Pierre Dillard, Henrique O. Duarte, Sarah Josefsson, Catarina Gomes, Constance de Montlaur, Henrik T.Z. Hellesøy, Ryan Dunkel, Erlend.B. Smeland, June H. Myklebust, Celso A. Reis, Emmet McCormack, Pascal Gelebart, Else Marit Inderberg, and Sébastien Wälchli

Figure S1. HH2 scFv exhibits very few mutations compared to germline sequence

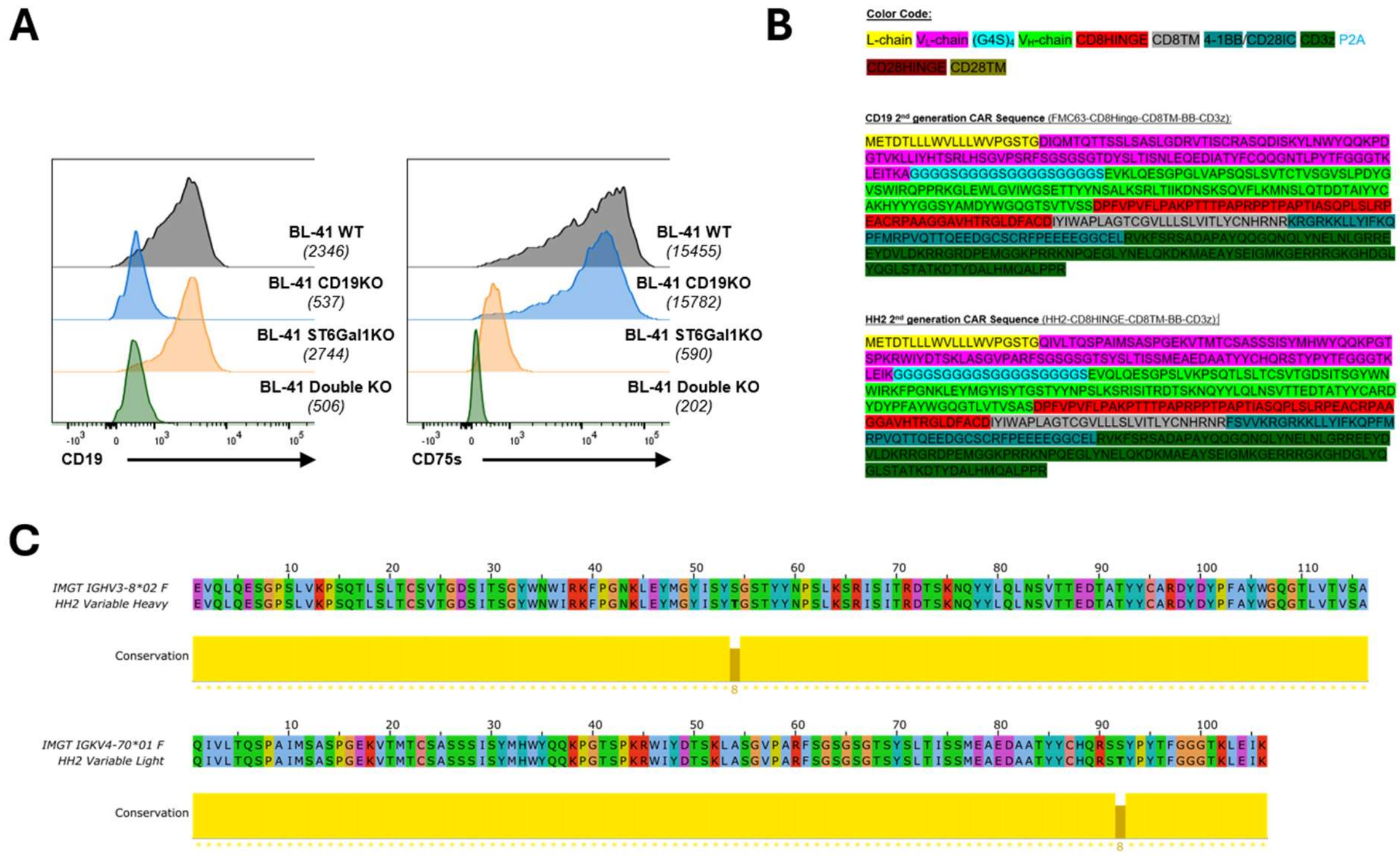

Figure S1. HH2 scFv exhibits very few mutations compared to germline sequence. (A) FCM staining of BL-41 either WT, CD19 KO, ST6GAL1 KO or double KO (representative for n=2 technical replicates). Left, CD19 staining. Right, HH2 staining. Median FI in parenthesis.

(B) Annotated sequence of the HH2 scFv.

(C) Alignment of amino acid sequences from the variable domains of HH2 with the germline counterparts identified on IMGT (IGHV3-8*02 F).

Figure S2. Combinatorial CAR Constructs

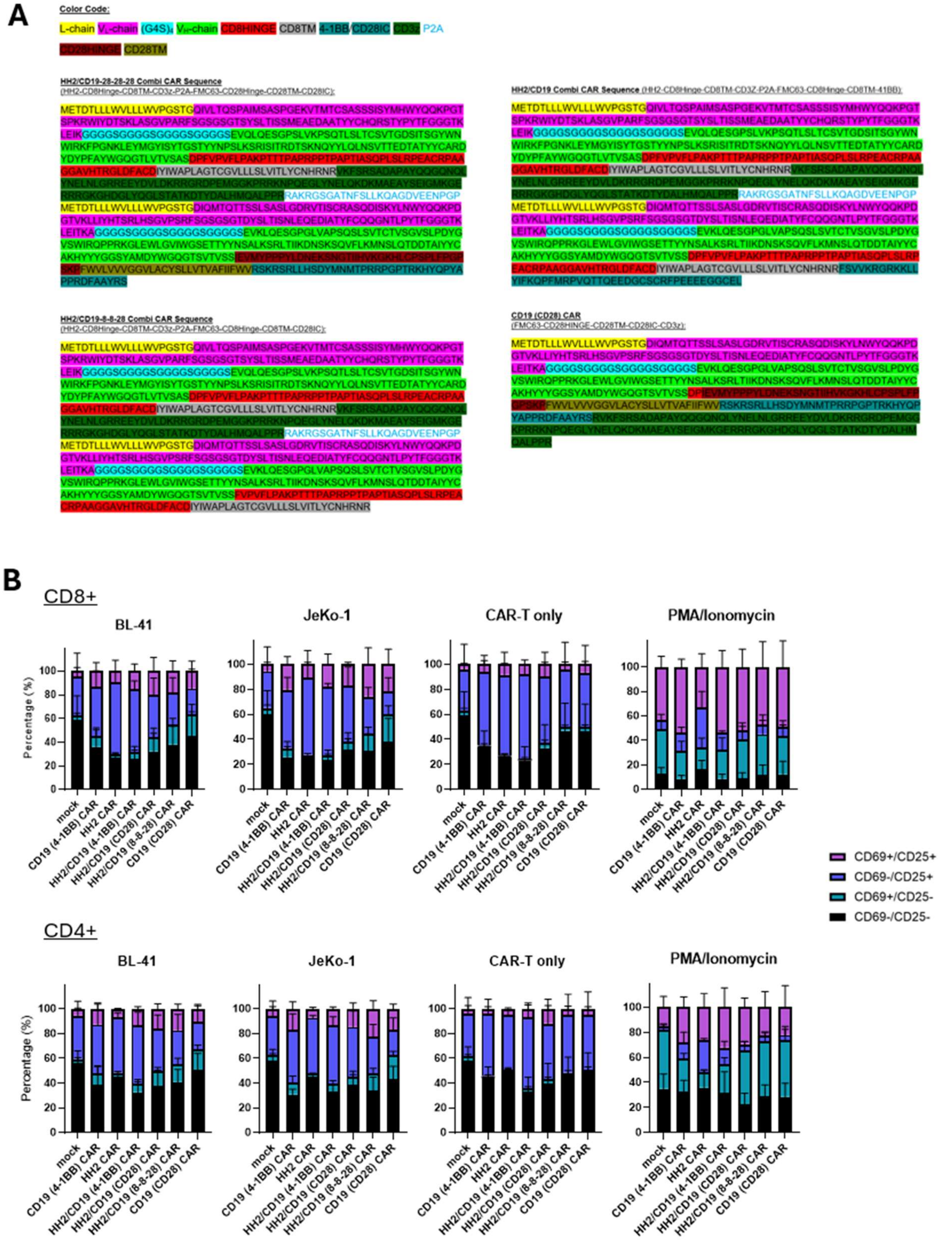

Figure S2. Combinatorial CAR Constructs. A) Annotated Sequences of the HH2/CD19 (CD28-CD28-CD28) CombiCAR, the HH2/CD19 (CD8-CD8-CD28) CombiCAR, the HH2/CD19 (4-1BB) CombiCAR, and the CD19 (CD28) CAR designs.

B) Percentage of CD25 and/or CD69 positive CD8+ T cells (top) or CD4+ T cells (bottom) after 24h co-culture with BL-41, JeKo-1, monoculture of CAR-T cells or addition of PMA and Ionomycin as positive control with N=3 healthy donors and n=2 technical replicates. Error bars are SD.
